# A new insight on copper: Promotion of collagen synthesis and myofiber growth and development in juvenile grass carp (*Ctenopharyngodon idella*)

**DOI:** 10.1016/j.aninu.2023.06.009

**Published:** 2023-07-08

**Authors:** Rui Ma, Lin Feng, Pei Wu, Yang Liu, Hong-Mei Ren, Shu-Wei Li, Ling Tang, Cheng-Bo Zhong, Dong Han, Wen-Bing Zhang, Jia-Yong Tang, Xiao-Qiu Zhou, Wei-Dan Jiang

**Affiliations:** a Animal Nutrition Institute, Sichuan Agricultural University, Chengdu, 611130, China; b Fish Nutrition and Safety Production University Key Laboratory of Sichuan Province, Sichuan Agricultural University, Chengdu, 611130, China; c Key Laboratory of Animal Disease-Resistance Nutrition, Ministry of Education, Ministry of Agriculture and Rural Affairs, Key Laboratory of Sichuan Province, Sichuan, 611130, China; d Animal Nutrition Institute, Sichuan Academy of Animal Science, Sichuan Animtech Feed Co. Ltd, Chengdu, 610066, Sichuan, China; e State Key Laboratory of Fresh Water Ecology and Biotechnology, Institute of Hydrobiology, Chinese Academy of Sciences, Wuhan, 430072, China; f The Key Laboratory of Mariculture, Ministry of Education, The Key Laboratory of Aquaculture Nutrition and Feeds, Ministry of Agriculture, Ocean University of China, Qingdao, 266003, China

**Keywords:** Copper, Grass carp (*Ctenopharyngodon idella)*, Collagen synthesis, Myofiber

## Abstract

Copper (Cu) is a trace element, essential for fish growth. In the current study, in addition to growth performance, we first explored the effects of Cu on collagen synthesis and myofiber growth and development in juvenile grass carp (*Ctenopharyngodon idella)*. A total of 1080 fish (11.16 ± 0.01 g) were randomly divided into 6 treatments (3 replicates per treatment) to receive five doses of organic Cu, which were Cu citrate (CuCit) at 0.99 (basal diet), 2.19, 4.06, 6.15, and 8.07 mg/kg, and one dose of inorganic Cu (CuSO_4_·5H_2_O at 3.15 mg/kg), for 9 weeks. The results showed appropriate Cu level (4.06 mg/kg) enhanced growth performance, improved nutritional Cu status, and downregulated Cu-transporting ATPase 1 mRNA levels in the hepatopancreas, intestine, and muscle of juvenile grass carp. Meanwhile, collagen content in fish muscle was increased after Cu intake, which was probably due to the following pathways: (1) activating CTGF/TGF-β1/Smads signaling pathway to regulate collagen transcription; (2) upregulating of La ribonucleoprotein domain family 6 (*LARP6)* mRNA levels to regulate translation initiation; (3) increasing proline hydroxylase, lysine hydroxylase, and lysine oxidase activities to regulate posttranslational modifications. In addition, optimal Cu group increased myofiber diameters and the frequency of myofibers with diameter >50 μm, which might be associated with upregulation of cyclin B, cyclin D, cyclin E, proliferating cell nuclear antigen, myogenic determining factor *(MyoD)*, myogenic factor 5, myogenin (*MyoG)*, myogenic regulatory factor 4 and myosin heavy chain (*MyHC)* and downregulation of myostatin mRNA levels, increasing protein levels of MyoD, MyoG and MyHC in fish muscle. Finally, based on percentage weight gain (PWG), serum ceruloplasmin (Cp) activity and collagen content in fish muscle, Cu requirements were determined as 4.74, 4.37 and 4.62 mg/kg diet (CuCit as Cu source) of juvenile grass carp, respectively. Based on PWG and Cp activity, compared to CuSO_4_·5H_2_O, the efficacy of CuCit were 131.80% and 115.38%, respectively. Our findings provide new insights into Cu supplementation to promote muscle growth in fish, and help improve the overall productivity of aquaculture.

## 1 Introduction

Copper (Cu), as a trace mineral element, is essential for several biological processes, including hemoglobin synthesis, bone formation, and myelin maintenance in the nervous system, and it also acts as a substance of key enzymes such as cytochrome oxidase and Cu–Zn superoxide dismutase enzyme (Gaetke et al., 2014). Cu is also crucial for fish growth (Lall, 2003). It has been shown that suitable Cu levels improve the growth performance of on-growing grass carp (*Ctenopharyngodon idella)* (Tang et al., 2013) and Indian juvenile carp (*Labeo rohita*) (Musharraf and Khan, 2022). Animal growth is primarily determined by skeletal muscle development, which is inseparable from myofiber growth and development, as well as extracellular matrix (ECM) deposition (Karalaki et al., 2009). Collagen is the most plentiful ECM element in skeletal muscle tissue (Mukund et al., 2017) and is essential for muscle development and structural stability (Csapo et al., 2020). A previous study showed that Cu deficiency led to impairment of collagen cross-linking and maturation in rat hearts (Werman and David, 1996). In addition, Cu had a certain effect on the proliferation and differentiation of mouse primary myoblasts (Vest et al., 2018). It was indicated Cu influenced collagen synthesis and myofiber growth and development. Nevertheless, to date, the effects of Cu on collagen synthesis and myofiber growth and development have not been reported in fish. Therefore, systematic, and in-depth research is essential.

Collagen synthesis in vertebrates generally involves transcription, translation, and posttranslational modifications (Yu et al., 2019). Collagen transcription and translation initiation are regulated by the transforming growth factor-β1 (TGF-β1)/Smads signaling pathway (Ma et al., 2020), and La ribonucleoprotein domain family 6 (LARP6) (Stefanovic et al., 2022), respectively. In addition, collagen posttranslational modifications are partially regulated by proline hydroxylase (PHD) (Yang et al., 2022), lysine hydroxylase (LH) (Wan et al., 2020) and lysine oxidase (LOX) (Trackman et al., 2015). Studies on LOX have been reported. It showed that Cu deficiency downregulated *LOX* mRNA levels in goat hearts, leading to impaired collagen cross-linking (Mandour et al., 2021). However, the regulation of collagen transcriptional, translational, and posttranslational modifications (except for LOX) by Cu have not been studied in animals. Cu is crucial for the transfer and metabolism of iron (Sharp, 2004). A study in our laboratory showed that suitable iron upregulated *TGF-β1* mRNA levels in grass carp skin (Guo et al., 2017). Meanwhile, suitable iron elevated PHD activity in mouse hepatoma cells (Martin et al., 2005). In addition, Cu upregulated insulin-like growth factor-1 (*IGF-1*) mRNA levels in human cardiomyocytes (Jiang et al., 2007), while *IGF-1* could upregulate *LARP6* mRNA levels in mouse smooth muscle (Blackstock et al., 2014). These findings implied that Cu might have an impact on collagen synthesis in animal muscle, which needs to be explored.

As an important component in the ECM, collagen enhances adhesion kinase activity and thus stimulates myofiber growth and development (Liu et al., 2020). Unlike terrestrial animals, the myofiber growth and development of fish mainly depends on proliferation and hypertrophy (Valente et al., 2013). Myofiber proliferation and hypertrophy are regulated by cell cycle proteins (such as cyclin B and cyclin D) (Mastroyiannopoulos et al., 2012) and myogenic determinants (such as myogenic determining factor (MyoD), myogenin (MyoG), and myosin heavy chain (MyHC)) (Zammit, 2017), respectively. Nevertheless, the effects and regulatory mechanisms of Cu on myofiber proliferation and hypertrophy have not been reported in animals. A previous study reported that Cu upregulated antioxidant-1 (*ATOX-1*) mRNA levels in finishing pig liver (Wen et al., 2022), and ATOX-1 upregulated cyclin B1 and cyclin D1 mRNA levels in mouse embryonic fibroblasts (Itoh et al., 2008). In addition, Cu downregulated Cu-transporting ATPase 1 (*ATP7A*) mRNA levels in finishing pig jejunal mucosa (Wen et al., 2022), and low levels of *ATP7A* upregulated *MyoD*, *MyoG* and *MyHC* mRNA levels in mouse myogenic cells (Abdelsaid et al., 2022; Gabay et al., 2020). Therefore, we speculated that Cu might promote skeletal muscle growth related to cell proliferation regulators and myogenic differentiation factors in fish, which needs to be studied.

In general, dietary Cu sources include inorganic (such as CuSO_4_·5H_2_O) and organic Cu in fish. Cu citrate (CuCit), as a new organic Cu source, has the advantages of good safety, low addition and emission, easy absorption, and high utilization rate in the animal body. CuCit can effectively provide Cu elements and protect the ecological environment (Yan et al., 2015). Grass carp is native to China and has been oriented to many countries, it is one of the most critical species for freshwater aquaculture (Wu et al., 2012). At present, the Cu requirements of grass carp have only been reported in our laboratory, and CuSO_4_·5H_2_O was used as the Cu source (Tang et al., 2013). However, different Cu sources have different Cu requirements. The Cu requirements of juvenile grouper (*Epinephelus malabaricus*) supplemented with CuSO_4_·5H_2_O (5.36 mg/kg) (Lin et al., 2008) was higher than that of Cu peptide (organic Cu, 2.19 mg/kg) based on weight gain (Lin et al., 2010). In addition, there are differences in the bioavailability of different Cu sources. Studies have shown that 125 mg/kg Cu (CuCit) and 250 mg/kg Cu (CuSO_4_) were equally effective in weaned pig (Armstrong et al., 2004) and broiler chicken (Pesti and Bakalli, 1996) growth. Therefore, the bioavailability of CuCit might be higher than that of CuSO_4_. However, no studies have evaluated dietary Cu requirements (CuCit as Cu source) and the efficacy of CuCit in comparison to CuSO_4_·5H_2_O of grass carp, which is crucial to investigate.

In summary, we first investigated the influences of Cu on collagen synthesis (except for LOX) and related signaling pathways, as well as on myofiber proliferation and hypertrophy in juvenile grass carp. The study might offer a rudimentary theoretical foundation for the regulation and mechanism of Cu on fish skeletal muscle growth. Cu requirements (CuCit as Cu source), and CuCit bioavailability relative to CuSO_4_·5H_2_O of juvenile grass carp were also determined to provide a reference for commercial formulation feed for fish.

## 2 Materials and methods

### 2.1 Animal ethics statement

All the procedures in this study were authorized by the Institutional Animal Care Advisory Committee of Sichuan Agricultural University (No. MR-2020314085).

### 2.2 Experimental design and diets

Table 1 displays the ingredients in the basal diet. Protein sources were mainly composed of casein, gelatin, and wheat gluten, while lipid sources were mainly consisted of fish oil and soybean oil. The basal diet was formulated to contain approximately crude protein at 324.34 g/kg diet and crude lipid at 47.87 g/kg diet, which were measured by the Kjeldahl method and the Soxhlet exhaustive extraction technique respectively using the standard methodology (AOAC, 1995). Calcium and total phosphorus were 5.27 and 15.48 g/kg diet, respectively, and were determined according to the national standard Determination of Calcium in Feed (GB/T 6436-2018) and Determination of total phosphorus in feed (GB/T 6437-2018), respectively. The crude fiber and gross energy were measured using a fiber analyzer (A2000I, ANKOM Technology, New York, USA) and oxygen bomb calorimeter (6400, Parr Instrument Company, Moline, USA), respectively, according to the method described by Zhang et al. (2016). The n-3 and n-6 PUFA were calculated referring to Zeng et al. (2016). The available phosphorus was calculated by NRC (National Research Council, 2011). Two Cu sources (Sichuan Animal Feed Co., Ltd.) were used: CuCit (Cu purity 34.5%) and CuSO_4_·5H_2_O (Cu purity 24.5%). The experiment used the following treatment structure: 1 (Cu-free control) + 4 (CuCit) +1 (CuSO_4_·5H_2_O). The Cu-free diet served as the control group, in which Cu content was at 0.96 mg/kg diet (calculated by measuring Cu contents in the ingredients with atomic absorption spectrometry). Cu was added to the test diets to provide graded Cu concentrations of 0.96 (un-supplemented control, Cu-deficient), 2.00, 4.00, 6.00, 8.00 and 3.00 mg/kg diet. The final Cu contents in the six treatments were determined to be 0.99 (un-supplemented control, Cu-deficient) and 2.19, 4.06, 6.15, 8.07 and 3.15 mg/kg diet. The diets were made employing the method described by Mai et al. (2009). In short, the ingredients were ground through a 300-μm sieve into a fine powder. The oil and water were added to the premixed dry ingredients, mixed well, squeezed through an extruder with a mold and air-dried at room temperature. According to Wang et al. (2016), the diets were broken up, sifted into pellets, and stored at −20 °C.

**Table 1:** Ingredients and nutrient composition of the basal diet (air-dry basis, g/kg).

Ingredients	Content	Nutrients	Content
Fish meal (CP, 67.10%)	80.00	n-3 PUFA^6^	10.40
Wheat gluten (CP, 71.53%)	80.00	n-6 PUFA^6^	9.60
Gelatin (CP, 90.18%)	100.00	Available phosphorus^7^	8.40
Amino acid mix^1^	123.30	Total phosphorus^8^	15.48
Fish oil	21.80	Crude protein^8^	324.34
Soybean oil	17.60	Crude lipid^8^	47.87
Corn starch	209.15	Calcium^8^	5.27
α-Starch	240.00	Crude fiber^8^	67.72
Microcrystalline cellulose	50.00	Gross energy^8^, MJ/kg	17.10
NaH_2_PO_4_	33.00		
Vitamin premix^2^	10.00		
Mineral premix (Cu-free)^3^	20.00		
Choline chloride^4^	10.00		
CuCit/CuSO_4_·5H_2_O premix^5^	5.00		
Butylated hydroxyanisole (BHA)	0.15		

CP = crude protein; CuCit = Cu citrate; PUFA = polyunsaturated fatty acids.

1 Provided the following per kilogram of amino acid mix: Lys, 1.22 g; Met, 0.55 g; Trp, 0.23 g; Thr, 0.88 g; Arg, 0.52 g; His, 0.54 g; Leu, 1.33 g; Ile, 0.80 g; Phe, 0.62 g; Tyr, 0.51 g; Val, 0.91 g, respectively.

2 Provided the following per kilogram of vitamin premix: retinyl acetate (1000,000 IU/g), 0.40 g; cholecalciferol (500,000 IU/g), 0.32 g; DL-α-tocopherol acetate (50%), 40.00 g; menadione (50%), 0.38 g; cyanocobalamin (1%), 0.94 g; D-biotin (2%), 1.55 g; folic acid (95%), 0.38 g; thiamine nitrate (98%), 0.13 g; ascorbic acid (95%), 16.32 g; niacin (99%), 2.58 g; inositol (97%), 22.06 g; calcium-D-pantothenate (98%), 3.85 g; riboflavin (80%), 0.78 g; pyridoxine hydrochloride (98%), 0.62 g. All ingredients were diluted with corn starch to 1 kg.

3 Provided the following per kilogram of mineral premix (Cu-free): MnSO_4_·H_2_O (31.8% Mn), 3.07 g; MgSO_4_·H_2_O (15.0% Mg), 237.83 g; FeSO_4_·H_2_O (30.0% Fe), 12.25 g; ZnSO_4_·H_2_O (34.5% Zn), 7.68 g; selenium yeast (0.2% Se) 13.65 g; Ca (IO_3_)_2_ (3.2% I), 1.56 g, and all ingredients were diluted with corn starch to 1 kg.

4 Provided the following per kilogram of choline chloride premix: choline chloride (50%), 306.71 g, and the rest was diluted with corn starch to 1 kg.

5 Povided the following per kilogram diet for the treatments: 0.99 (un-supplemented) and 2.19, 4.06, 6.15, 8.07 and 3.15 mg/kg, and the rest was diluted with microcrystalline cellulose.

6 n-3 PUFA, and n-6 PUFA were calculated by NRC (2011) contents were referenced to Zeng et al. (2016).

7 Available phosphorus was calculated according to NRC (National Research Council) (2011).

8 Crude protein, crude lipid, total phosphorus, calcium contents, crude fiber and gross energy were measured value.

### 2.3 Feeding management

A local farm provided juvenile grass carp (Chengdu, China). Experimental fish were reared in outdoor freshwater ponds and fed five times per day (08:00, 11:00, 13:00, 15:00 and 19:00). Prior to the trial, they were fed a commercial diet at a rate of 4% of initial body weight for 4 weeks to acclimatize to the experimental environment and were then fed a basal diet (Cu-free) for 2 weeks to decrease Cu content according to Tang et al. (2013). Later, a total of 1080 fish were randomly divided into 18 net cages (150 cm × 150 cm × 150 cm, 60 fish per cage) with an initial mean weight of 11.16 g/fish, and divided into six treatments, each with three replicates. As described by Wu et al. (2017), we fitted a disc with a diameter of 80 cm to the bottom of each cage and wrapped the disc with 1-mm gauze to collect the remaining feed. During a 9-week experiment, the corresponding experimental feed was fed until saturated. After feeding for 30 min, the uneaten feed was siphoned out, dried, and weighed. Feed intake was calculated on a dry matter basis according to the method described by Cai et al. (2005). For culture water impurity removal and ammonia concentration reduction, water was pumped through a sand filter at a rate of 1 L/min in each cage according to Wu et al. (2017) method. Throughout the trial period, microporous aeration was used, and water was changed daily. We used a professional multiparameter instrument (YSI Incorporated, Yel-low Springs, OH, USA) to measure water quality every day. Water temperature and pH value were determined at 27.8 ± 3 °C and 7.5 ± 0.4, respectively, and dissolved oxygen was ≥6.0 mg/L. Cu concentration in the culture water was determined to be 5 μg/L in accordance with the Standard test methods for drinking water - metal indicators (GB/T 5750.6-2006).

### 2.4 Sample collection and analysis

After the feeding trial, all fish were starved for 24 h and given a benzocaine bath (50.0 mg/L) for anesthesia. At the start of sampling, the body weight, length, width, and height were measured prior to blood collection. Then, blood was collected from the tail vein of fish, centrifuged, and the supernatant was removed and stored at −80 °C according to Wang et al. (2015). Fish were killed to collect samples in the hepatopancreas, intestine and muscle. Tissue samples for body composition, Cu contents, enzyme activities, mRNA and protein level analysis were frozen with liquid N_2_ and then kept at −80 °C. Muscle samples for sectioning analysis were stored in 4% para-formaldehyde.

The approximate composition of whole fish and muscle were determined using the standard methodology (AOAC, 1995). The cooking loss, pH value and shear force of muscle were determined by applying Wang et al. (2015) method. Hydroxyproline (HYP) content was analyzed with kits obtained from China's Nanjing Jiancheng Bioengineering Research Institute. The collagen content was calculated by multiplying the HYP content by 8 based on the approach of Sun et al. (2017).

### 2.5 Biochemical analysis

Cu contents in hepatopancreas, serum and muscle were determined by atomic absorption spectrometry (CONTAA700, Jena Analytical Instruments AG, Germany) according to *National Standard for Safety Determination of Cu in Food* (GB 5009.13-2017). Serum ceruloplasmin (Cp) activity was analyzed with kits obtained from China's Nanjing Jiancheng Bioengineering Research Institute, which was measured using o-dianisidine dihydrochloride as a substrate. Muscle tissue was weighed 100 mg, homogenized in 0.9% sterile saline (1:10, wt/vol), subsequently incubated on ice for 30 min and centrifuged at 6000 × *g* for 20 min at 4 °C to collect the supernatant to obtain a muscle tissue homogenate, which was used to measure muscle enzyme activities (Wang et al., 2015). Enzyme activities such as PHD, LH and LOX were measured using ELISA kits (Shanghai Changjin Biotechnology Co., Ltd, Shanghai, China).

### 2.6 Histological analysis

Muscle samples were routinely paraffin embedded and sectioned after being treated with 4% paraformaldehyde. Hematoxylin-eosin (H&E) staining was used to stain 4 μm paraffin sections. ImageJ software was used to analyze the myofiber mean diameters of three images (for each picture, 300 myofibers were chosen at random). The frequency of various myofiber sizes (<20, 20 to 50, and >50 μm) was determined employing the method of Valente et al. (2016).

Adopting the method of Lin et al. (2022), using Sirius red staining, the collagen fiber area was examined in grass carp muscle. Briefly, muscle paraffin slices (4 μm) were dewaxed, rehydrated, and then stained with Sirius red solution sets (1 g/L Sirius red F3B in picric acid for 90 min, and 0.01 M HCl for 10 min, respectively). The collagen region was evaluated using ImageJ software (3 complete images for each group; red; %). All sections were observed using an upright light microscope (Japan, Nikon).

### 2.7 Real-time PCR analysis

The real-time PCR procedure was performed using the method of Ma et al. (2020) in our laboratory. In brief, an RNA iso Plus kit (Dalian Takara, China) was used to isolate total RNA from hepatopancreas, intestinal and muscle tissues. Next, total RNA was treated with DNAse I. RNA quality and quantity were determined by electrophoresis on 1% agarose gels and Nanodrop 2000 (Thermo Fisher Scientific, Wilmington, DE, USA), respectively. Additionally, the PrimeScript RT kit was used to reverse transcribe RNA to cDNA. Primer 5.0 software was used to build the primers using the Sus-Scrofa sequences in the NCBI database and the primers were synthesized by Bioengineering (Shanghai) Co., Ltd. The primers are listed in Table S1. Beta-actin was used as an endogenous control based on selection. The gene expression results were measured by the 2^−ΔΔCt^ method.

### 2.8 Western blotting

The previous method described by Dong et al. (2018) was adopted to analyze Western blotting. Briefly, RIPA lysis buffer (Chinese Beyotime) was used to extract total muscle protein, and then protein concentrations were measured using a BCA assay kit (Beyotime Biotechnology Inc., China). Then, SDS-polyacrylamide gel electrophoresis (SDS-PAGE) was applied, and the target proteins (40 μg per sample) were separated and transferred to polyvinylidene-fluoride (PVDF) membranes. The PVDF membranes were blocked for 2 h at room temperature, followed by overnight incubation with primary antibodies (TGF-β1, Col1α1, Col1α2, total-Smad2, p-Smad2 Ser467, Smad3, Smad4, MyoD, MyoG, MyHC and β-actin) at 4 °C. These antibodies were purchased from Ebtek Biotechnology Co., Ltd. The following day, the blots were washed 3 times and then incubated with the enzyme-labeled secondary antibody (goat anti-rabbit, Santa Cruz Biotechnology, Santa Cruz, CA, USA) in Tris Buffered Saline with Tween 20 for 2 h. Finally, Enhanced chemiluminescence kit (Beyotime Biotechnology, Inc., China) was used for imaging, and Image Lab 6.1 was used to view the immunological complex.

### 2.9 Calculating and statistical analyses

Table 2 summarizes the formulas of the growth performance indices. The mean ± standard deviation (SD) was used to express the results. SPSS software was used to conduct statistical analysis. Using one-way ANOVA and Duncan's multiple range test, the Cu-deficient and CuCit groups were compared. Then, an independent sample *t*-test was used for Cu-deficient group and CuSO_4_·5H_2_O group, and an independent sample *t*-test was used for CuCit optimal group and CuSO_4_·5H_2_O group. Significance was considered with *P* < 0.05. We conducted unary linear regression, quadratic regression, and triple regression for comparison to predict a more accurate response to the dietary intake based on the results of *R*^2^ and *P-*value using a quadratic regression model to calculate the dietary Cu requirements of the fish.

**Table 2:** Index formulas for growth performance of juvenile grass carp (*Ctenopharyngodon idella*).

Item	Formulas
PWG	PWG (%) = [(FBW, g/fish) - (IBW, g/fish)]/(IBW, g) × 100
SGR	SGR (%/d) = [ln (FBW, g/fish) - ln (IBW, g/fish)]/d × 100
FE	FE = [(FBW, g/fish) - (IBW, g/fish)]/(FI, g/fish)
CF	CF (g/cm^3^) = (body weight, g)/(body length^3^, cm^3^) × 100
Survival rate	Survival rate (%) = final fish number/initial fish number × 100
PPV	PRV (%) = (fish protein gain, g)/(protein intake, g) × 100
LPV	LRV (%) = (fish lipid gain, g)/(lipid intake, g) × 100

IBW = initial body weight; FBW = final weight; FI = feed intake; PWG = percentage weight gain; FE = feed efficiency; SGR = specific growth rate; CF = condition factor; PPV = protein production value; LPV = lipid production value.

## 3 Results

### 3.1 Growth performance, Cu status and chemical composition in juvenile grass carp

Table 3 lists the growth parameters. There was no significant difference in the mean initial body weight of grass carp among all groups (*P* > 0.05). With increasing Cu levels up to 4.06 mg/kg, the final body weight (FBW), percentage weight gain (PWG), specific growth rate (SGR), feed intake (FI), and feed efficiency (FE), total length, body length, width and height were significantly higher than those of the Cu-deficient group (*P* < 0.05). The total length, width, and height of the body at the 4.06 mg/kg CuCit level were significantly higher than those at the CuSO_4_·5H_2_O level (*P* < 0.05). In addition, condition factor (CF) increased significantly (*P* < 0.05) as the Cu levels increased to 2.19 mg/kg compared to the Cu deficient group. Nevertheless, Cu did not affect the survival rate of juvenile grass carp (*P* > 0.05). With the enhancement of Cu levels, serum Cp activity rose sharply (*P* < 0.05) and peaked at 4.06 mg/kg Cu level. At the same time, Cp activity was notably higher at 4.06 mg/kg CuCit in comparison to the group of inorganic CuSO_4_·5H_2_O (*P* < 0.05).

**Table 3:** Effects of diets containing graded levels of Cu on the growth performance of juvenile grass carp (*Ctenopharyngodon idella*).

Item	Dietary Cu level (mg/kg diet)						*P*-value
	CuCit					CuSO_4_·5H_2_O	
	0.99	2.19	4.06	6.15	8.07	3.15	
IBW^1^, g/fish	11.17 ± 0.02	11.16 ± 0.01	11.16 ± 0.01	11.18 ± 0.02	11.16 ± 0.001	11.17 ± 0.03	0.210
FBW^1^, g/fish	105.54 ± 1.86^a^	140.57 ± 0.57^c^	172.65 ± 0.84^e^	147.12 ± 1.07^d^	124.76 ± 0.61^b^	143.71 ± 1.00∗^#^	<0.001
FI^1^, g/fish	101.04 ± 0.37^a^	123.48 ± 0.11^c^	144.62 ± 0.06^e^	126.06 ± 0.08^d^	109.50 ± 0.10^b^	125.34 ± 0.17	<0.001
WG^1^, g/fish	94.37 ± 1.85^a^	129.42 ± 0.57^c^	161.49 ± 0.82^e^	135.94 ± 1.07^d^	113.60 ± 0.61^b^	132.53 ± 1.02∗	<0.001
PWG^1^, %	844.67 ± 15.49^a^	1159.95 ± 5.50^c^	1447.35 ± 5.67^e^	1216.00 ± 19.69^d^	1018.38 ± 5.57^b^	1186.23 ± 11.45∗^#^	<0.001
SGR^1^, %/d	3.56 ± 0.03^a^	4.02 ± 0.01^c^	4.35 ± 0.01^e^	4.09 ± 0.01^d^	3.83 ± 0.01^b^	4.05 ± 0.01∗^#^	<0.001
FE^1^	0.93 ± 0.02^a^	1.05 ± 0.01^c^	1.12 ± 0.01^d^	1.09 ± 0.03^c^	1.04 ± 0.01^b^	1.06 ± 0.01b∗^#^	<0.001
FCR^1^	1.07 ± 0.02^c^	0.95 ± 0.01^b^	0.90 ± 0.01^a^	0.92 ± 0.02^a^	0.96 ± 0.01^b^	0.95 ± 0.01∗^#^	<0.001
Survival rate^1^, %	99.44 ± 0.96	100.00 ± 0.00	100.00 ± 0.00	100.00 ± 0.00	100.00 ± 0.00	100.00 ± 0.00	0.452
CF^2^, g/cm^3^	1.26 ± 0.09^a^	2.72 ± 0.34^c^	1.85 ± 0.04^b^	1.85 ± 0.11^b^	1.75 ± 0.12^b^	1.87 ± 0.05∗	<0.001
Total length^2^, cm	19.62 ± 1.22^a^	22.00 ± 0.48^b^	25.15 ± 0.44^d^	23.48 ± 0.31^c^	21.52 ± 0.23^b^	22.97 ± 0.14∗^#^	<0.001
Body length^2^, cm	16.94 ± 0.91^a^	19.07 ± 0.48^b^	22.11 ± 0.26^d^	20.38 ± 0.42^c^	18.88 ± 0.58^b^	19.54 ± 0.16∗^#^	<0.001
Body width^2^, cm	2.09 ± 0.17^a^	2.32 ± 0.04^b^	2.93 ± 0.04^d^	2.60 ± 0.03^c^	2.35 ± 0.05^b^	2.46 ± 0.01^#^	<0.001
Body height^2^, cm	3.14 ± 0.19^a^	3.62 ± 0.04^bc^	4.34 ± 0.06^d^	3.85 ± 0.14^c^	3.46 ± 0.15^b^	3.66 ± 0.06^#^	<0.001
Ceruloplasmin^3^, U/L	12.31 ± 1.03^a^	14.13 ± 0.77^b^	18.43 ± 1.36^c^	15.15 ± 0.82^b^	14.67 ± 1.05^b^	15.58 ± 1.18∗^#^	<0.001

CuCit = Cu citrate; IBW = initial body weight; FBW = final body weight; FI = feed intake; FE = feed efficiency; WG = weight gain; PWG = percent weight gain; SGR = specific growth rate; FCR = feed coefficient ratio; CF = condition factor.

Mean values within a row with different superscript letters indicate significant difference (one-way ANDOVA and Duncan's multiple-range tests at *P* < 0.05.

The asterisk (∗) indicates a significant difference between 0.99 mg/kg CuCit and CuSO_4_·5H_2_O groups (*P* < 0.05); the number sign (^#^) indicates a significant difference between 4.06 mg/kg CuCit and CuSO_4_·5H_2_O groups (*P* < 0.05).

1 Values are means ± SD, *n* = 3 (for 3 replicate groups, 60 fish per replicate).

2 Values are means ± SD, *n* = 3 (for 3 replicate groups, 6 fish per replicate).

3 Values are means ± SD, *n* = 3 (for 3 replicate groups, 2 fish per replicate).

**Fig. 1:**
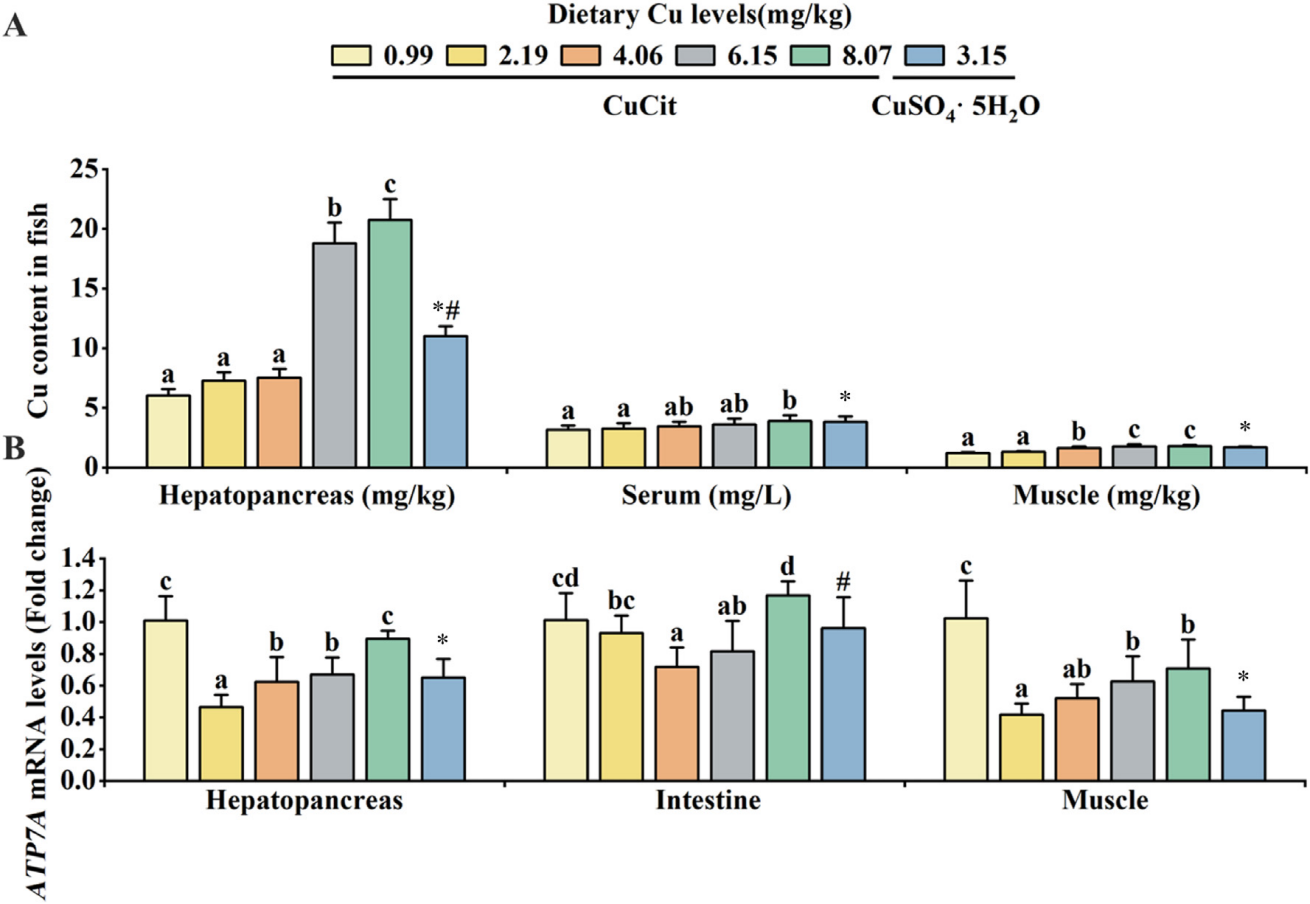
Effects of different Cu levels on Cu content in hepatopancreas, serum and muscle (A) and the Cu transporter *ATP7A* mRNA expression (B) in hepatopancreas, intestine and muscle of juvenile grass carp. Mean values with different superscript letters indicate significant difference (one-way ANOVA and Duncan's multiple-range tests, *P* < 0.05). The asterisk (∗) indicates a significant difference between 0.99 mg/kg CuCit and CuSO_4_·5H_2_O groups (*P* < 0.05); the number sign (^#^) indicates a significant difference between 4.06 mg/kg CuCit and CuSO_4_·5H_2_O groups (*P* < 0.05). Values are means ± SD, *n* = 3 (for 3 replicate groups, 2 fish per replicate). *ATP7A* = Cu-transporting ATPase 1.

The effects of Cu on the hepatopancreas, serum and muscle Cu contents in juvenile grass carp are shown in Fig. 1. As shown in Fig. 1A, the hepatopancreas, serum and muscle Cu contents increased linearly with increasing Cu levels, which considerably increased at the 8.07 mg/kg Cu level in comparison to the Cu-deficient group (*P* < 0.05). Fig. 1B depicts the effects of Cu on juvenile grass carp hepatopancreas, intestine, and muscle *ATP7A* mRNA levels, which were markedly reduced with dietary Cu levels reaching 4.06 mg/kg (*P* < 0.05). At the same time, the *ATP7A* mRNA level in the intestine was lower in the 4.06 mg/kg CuCit group than in the CuSO_4_·5H_2_O group (*P* < 0.05).

The effects of Cu on the whole-body and muscle nutritional value of juvenile grass carp are shown in Table 4. When fish were fed the diet with 4.06 mg/kg Cu level, the protein and lipid contents in the whole body reached the highest value and dropped as Cu levels increased (*P* < 0.05). With the enhancement of Cu levels, both protein and lipid production values (PPV, LPV) had a significant effect, which significantly increased before 4.06 mg/kg Cu level (*P* < 0.05) and then declined dramatically at higher levels (*P* < 0.05). In contrast, whole-body moisture reached its lowest value at 4.06 mg/kg Cu level. Moreover, both crude protein and lipid contents in fish muscle increased significantly with increasing Cu levels (*P* < 0.05), and the maximum value were 21.64 and 3.42 at 4.06 mg/kg Cu level, respectively. Juvenile grass carp muscle moisture was unaffected by Cu.

**Table 4:** Effects of diets containing graded levels of Cu on the proximate composition and physicochemical properties of the muscle of juvenile grass carp (*Ctenopharyngodon idella*).

Item	Dietary Cu level (mg/kg diet)						*P*-value
	CuCit					CuSO_4_·5H_2_O	
	0.99	2.19	4.06	6.15	8.07	3.15	
Whole body							
Moisture, %	75.80 ± 0.87^b^	75.38 ± 0.90^b^	72.04 ± 0.86^a^	72.45 ± 0.96^a^	73.10 ± 1.44^a^	72.25 ± 1.47	<0.001
Crude protein, %	16.39 ± 1.11^a^	18.53 ± 1.33^b^	24.14 ± 1.81^d^	20.70 ± 1.45^c^	18.37 ± 1.63^b^	21.36 ± 2.04∗^#^	<0.001
Crude lipid, %	7.09 ± 0.26^a^	7.68 ± 0.58^b^	9.76 ± 0.45^c^	7.95 ± 0.37^b^	7.74 ± 0.67^b^	8.66 ± 0.39∗^#^	<0.001
LPV, %	135.37 ± 4.29^a^	168.93 ± 8.93^b^	234.93 ± 16.42^c^	172.95 ± 2.42^b^	169.82 ± 7.94^b^	185.76 ± 8.52∗^#^	<0.001
PPV, %	47.19 ± 4.96^a^	61.21 ± 4.12^b^	88.23 ± 3.71^d^	70.88 ± 5.73^c^	61.23 ± 4.28^b^	74.72 ± 7.28∗^#^	<0.001
Muscle							
Moisture, %	76.78 ± 1.58	76.17 ± 1.18	74.03 ± 3.15	75.34 ± 2.96	75.92 ± 1.96	74.98 ± 2.60	0.326
Crude protein, %	16.61 ± 1.14^a^	18.24 ± 1.34^ab^	21.64 ± 2.12^c^	19.67 ± 1.37^b^	18.42 ± 1.63^b^	20.03 ± 0.63∗	<0.001
Crude lipid, %	2.12 ± 0.16^a^	2.19 ± 0.24^a^	3.42 ± 0.27^c^	2.85 ± 0.16^b^	2.66 ± 0.25^b^	2.85 ± 0.28∗^#^	<0.001
Cooking loss, %	21.31 ± 1.95^b^	17.26 ± 1.67^a^	17.26 ± 1.62^a^	19.83 ± 1.25^ab^	20.35 ± 1.73^ab^	18.66 ± 1.70	0.041
Shear force, N	1.21 ± 0.08^a^	1.83 ± 0.03^c^	2.71 ± 0.16^e^	2.18 ± 0.04^d^	1.54 ± 0.03^b^	2.22 ± 0.08∗^#^	<0.001
pH _24h_	6.24 ± 0.01^a^	6.34 ± 0.01^c^	6.53 ± 0.02^e^	6.44 ± 0.02^d^	6.29 ± 0.02^b^	6.37 ± 0.00∗^#^	<0.001
HYP, μg/mg	0.69 ± 0.06^a^	0.73 ± 0.06^ab^	0.89 ± 0.04^c^	0.77 ± 0.04^b^	0.71 ± 0.06^ab^	0.77 ± 0.05∗^#^	<0.001

CuCit = Cu citrate; LPV = lipid production value; PPV = protein production value; HYP = hydroxyproline.

^a-^^e^ mean values within a row with different superscript letters indicate significant difference (one-way ANOVA and Duncan's multiple-range tests, *P* < 0.05).

The asterisk (∗) indicates a significant difference between 0.99 mg/kg CuCit and CuSO_4_·5H_2_O groups (*P* < 0.05); the number sign (^#^) indicates a significant difference between 4.06 mg/kg CuCit and CuSO_4_·5H_2_O groups (*P* < 0.05).

Values are means ± SD, *n* = 3 (for 3 replicate groups, 2 fish per replicate).

The cooking loss decreased significantly with Cu levels rising to 4.06 mg/kg (*P* < 0.05), which at 4.06 mg/kg CuCit level was lower than CuSO_4_·5H_2_O group. The shear force, pH and HYP content reached a maximum at 4.06 mg/kg CuCit and their values at 4.06 mg/kg were higher than those of the CuSO_4_·5H_2_O group (*P* < 0.05).

### 3.2 Collagen-associated indicators in juvenile grass carp muscle

**Fig. 2:**
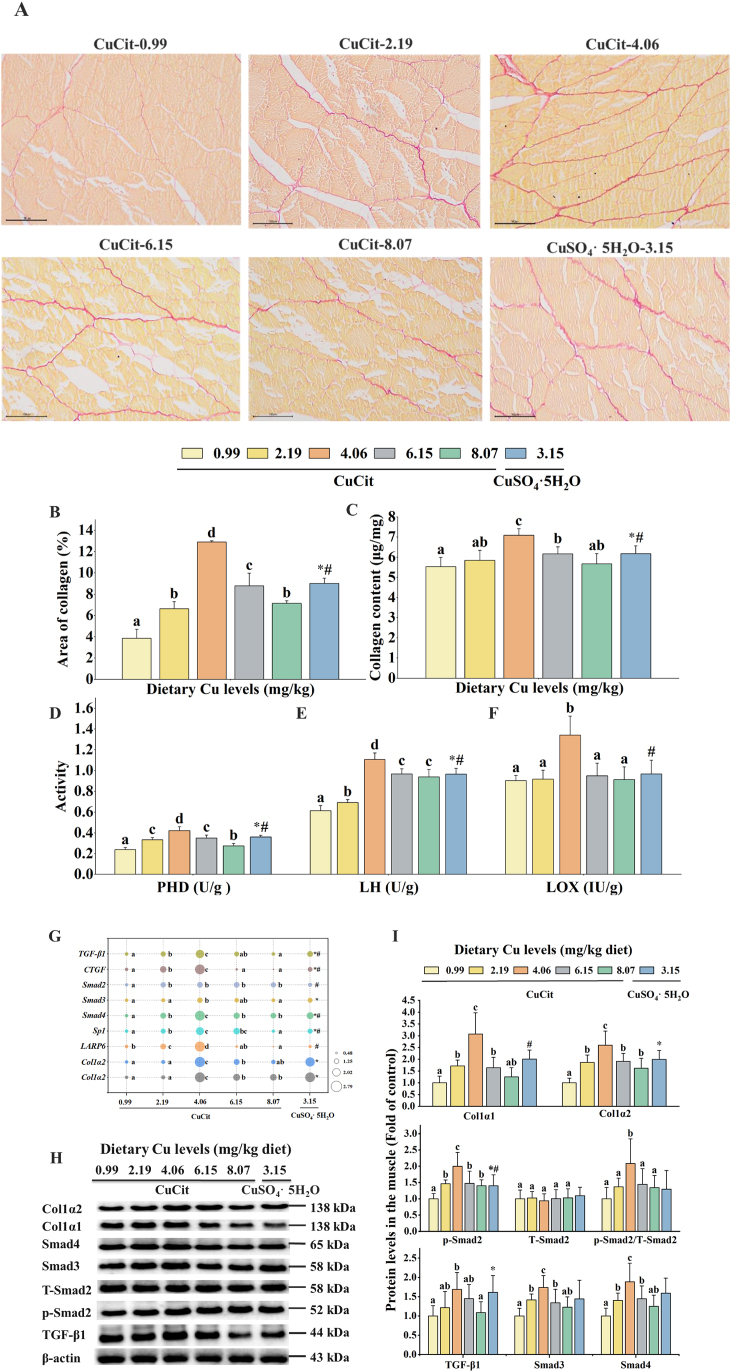
Effects of different Cu levels on collagen deposition in muscle of juvenile grass carp. (A) Sirius red staining of muscle tissue in fish (magnification 200× ; scale bar = 100 μm). Collagen was stained by red color (*n* = 3). (B) The proportion of collagen fibers according to statistical analysis of the Sirius red staining (*n* = 3). (C) Collagen content calculated from hydroxyproline content. (D) Proline hydroxylase (PHD; U/g tissue). (E) Lysine hydroxylase (LH; U/g tissue). (F) Lysine oxidase (LOX; IU/g tissue). (G) Relative mRNA expression. *Col1α1* = type I collagen α1; *Col1α2* = type I collagen α2; *LARP6* = La Ribonucleoprotein 6; *Sp1* = specificity protein 1; *CTGF* = connective tissue growth factor; *TGF-β1* = transforming growth factor-β1. (H–I) Western blot analysis of collagen synthesis in the muscle of juvenile grass carp fed diets containing different levels of Cu for 9 weeks. The fold changes are based on the mRNA levels of different genes. Mean values with different superscript letters indicate significant difference (one-way ANOVA and Duncan's multiple-range tests, *P* < 0.05. The asterisk (∗) indicates a significant difference between 0.99 mg/kg CuCit and CuSO_4_·5H_2_O groups (*P* < 0.05); the number sign (^#^) indicates a significant difference between 4.06 mg/kg CuCit and CuSO_4_·5H_2_O groups (*P* < 0.05). Values are means ± SD, *n* = 3 (for 3 replicate groups, 2 fish per replicate).

Fig. 2 depicts that Cu affects the collagen-related indicators in juvenile grass carp. Sirius red staining was used to analyze the effect of Cu levels on collagen deposition in juvenile grass carp muscle (Fig. 2A–B). The results demonstrated that the collagen fiber area (red) increased with increasing Cu levels when compared to the Cu-deficient group, reaching a maximum at 4.06 mg/kg (*P* < 0.05). Furthermore, the 4.06 mg/kg CuCit group was notably higher than the CuSO_4_·5H_2_O group (*P* < 0.05).

As shown in Fig. 2C–F, compared with the Cu-deficient group, the collagen content and PHD, LH and LOX activities in added Cu treatments were significantly increased (*P* < 0.05) and maximized at 4.06 mg/kg level. In addition, these indices in the 4.06 mg/kg CuCit level were higher than those in the CuSO_4_·5H_2_O group.

As shown in Fig. 2G–H, with dietary Cu rising to 4.06 mg/kg, the mRNA abundance of *Col1α1*, *Col1α2*, *TGF-β1*, *Smad3*, *Smad4*, *LARP6*, *Sp1*, and *CTGF*, as well as p-Smad2, Smad3, Smad4, Col1α1 and Col1α2 protein levels were markedly elevated (*P* < 0.05). When Cu levels rose to 2.19 mg/kg diet, the mRNA abundance of *Smad2* was significantly elevated (*P* < 0.05) and then plateaued. The T-Smad2 protein level was not significantly different among all groups (*P* > 0.05). At the same time, in the 4.06 mg/kg CuCit group, *TGF-β1*, *CTGF*, *Smad4*, *Sp1* and *LARP6* mRNA levels, and p-Smad2 protein levels were higher than those in the CuSO_4_·5H_2_O group.

### 3.3 Myofiber growth and development-associated indicators in juvenile grass carp muscle

**Fig. 3:**
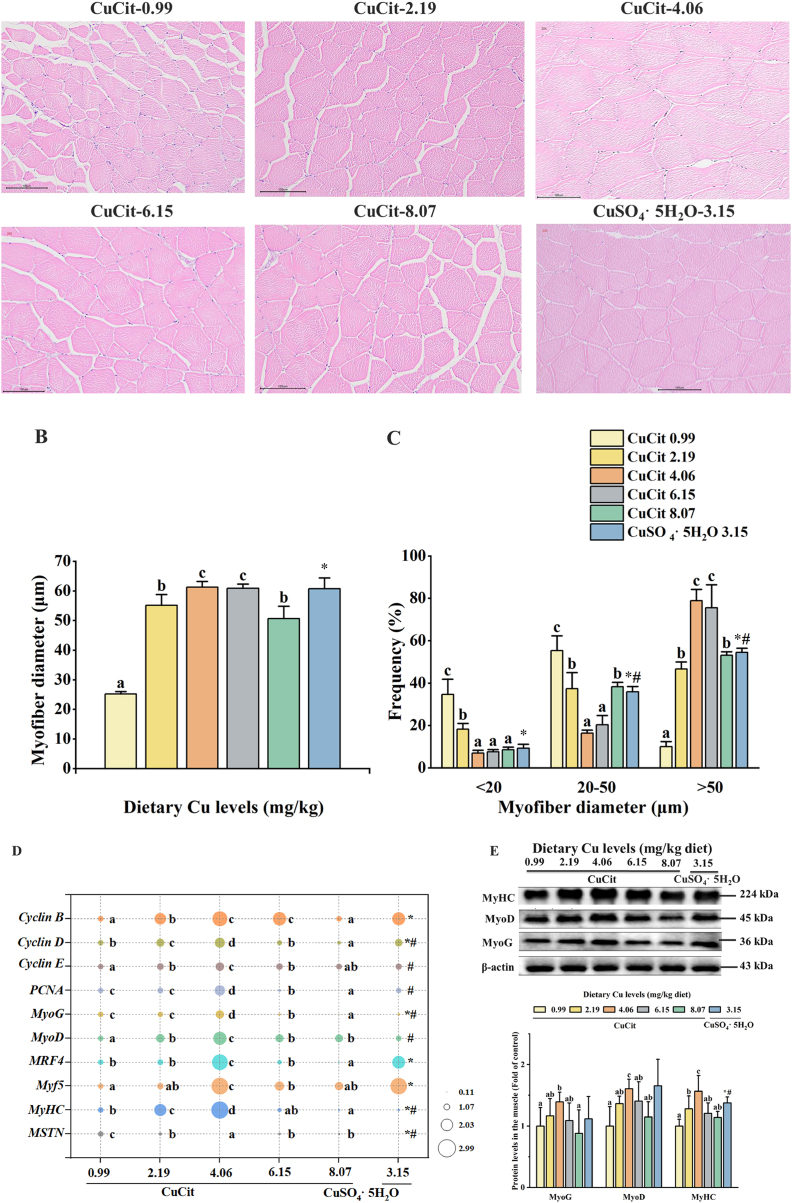
Effects of different Cu levels on the growth and development of myofiber in juvenile grass carp muscle. (A) Transversal section micro-structure (magnification 200× , scale bar = 100 μm) of muscle in juvenile grass carp supplemented with different Cu levels for 9 weeks visualized using H&E staining. (B) Myofiber diameters (*n* = 3). (C) Frequency of distribution (%) of the diameters of myofiber (*n* = 3). (D) Relative mRNA expressions and (E) Western blot analysis of myogenic regulatory factors in the muscle of juvenile grass carp fed diets containing different Cu levels for 9 weeks. The fold changes are based on the mRNA expression levels of different genes. Mean values with different superscript letters indicate significant difference (one-way ANOVA and Duncan's multiple-range tests, *P* < 0.05). The asterisk (∗) indicates a significant difference between 0.99 mg/kg CuCit and CuSO_4_·5H_2_O groups (*P* < 0.05); the number sign (^#^) indicates a significant difference between 4.06 mg/kg CuCit and CuSO_4_·5H_2_O groups (*P* < 0.05). Values are means ± SD, *n* = 3 (for 3 replicate groups, 2 fish per replicate).

Fig. 3A–C illustrates that Cu affects the myofiber characteristics of juvenile grass carp. The myofiber diameters of the 2.19, 4.06, 6.15 and 8.07 mg/kg Cu groups were considerably higher than those of the Cu-deficient group, and the frequency of myofibers >50 μm diameter showed the same trend as it (*P* < 0.05). Compared to the Cu-deficient group, the frequency of <20 and 20–50 μm diameter were markedly reduced in the other groups (*P* < 0.05).

Fig. 3D–E shows the effects of Cu on mRNA and protein levels associated with proliferation and differentiation of myogenic cells in juvenile grass carp muscle. Compared to the Cu-deficient group, the mRNA levels of cyclin B, cyclin D, cyclin E, proliferating cell nuclear antigen (*PCNA*)*, MyoG*, *MyoD*, *Myf5*, *MRF4* and *MyHC* at 4.06 mg/kg Cu level were significantly increased (*P* < 0.05) and then progressively declined. In contrast, as Cu levels increased, significantly lowered *MSTN* mRNA levels were observed (*P* < 0.05) and reached the lowest value at 4.06 mg/kg Cu level, and then steadily rose. Additionally, the protein levels of MyoG, MyoD, and MyHC increased significantly with Cu levels reaching 4.06 mg/kg diet (*P* < 0.05).

## 4 Discussion

### 4.1 Cu improved growth performance and Cu nutritional status of fish

This study showed that growth performance of juvenile grass carp was significantly influenced by appropriate dietary Cu. According to our findings, the PWG, FI, and FE of juvenile grass carp were increased by appropriate dietary Cu, proving that appropriate Cu level (4.06 mg/kg) could enhance the growth performance of juvenile grass carp, which was consistent with on-growing grass carp (Tang et al., 2013) and Indian juvenile carp (Musharraf and Khan, 2022).

Nutrient status in the fish influences their growth (Wei et al., 2018). Serum and tissue Cu levels sensitively reflect Cu nutritional status (Shao et al., 2010). Serum Cp, a member of the poly Cu oxidase family, is an enzyme with oxidase activity (Hellman and Gitlin, 2002). Serum Cp is also a good indicator of Cu nutritional status (Merle et al., 2009). Our findings demonstrated that supplemental Cu raised the hepatopancreas, serum, and muscle Cu concentrations, as well as serum Cp activity of juvenile grass carp, demonstrating that dietary Cu could improve Cu nutritional status, which might be related to ATP7A. ATP7A belongs to the Cu-ATPase P_18_ family and plays a vital role in the regulation of both cellular and systemic Cu homeostasis, which can transfer Cu from intracellular to extracellular (Lutsenko et al., 2007). Our findings demonstrated that appropriate Cu level decreased *ATP7A* mRNA abundance in the hepatopancreas, intestine, and muscle. The same results were obtained in finishing pigs (Wen et al., 2022). The outcomes listed above showed that cellular Cu export demand was reduced at optimal Cu concentrations, regulating cellular Cu flux, and thus maintaining Cu homeostasis.

Fish growth depends on nutrient deposition (Hua et al., 2007). Our results showed that suitable Cu level could increase the protein and lipid contents and PRV as well as LRV of the whole body in juvenile grass carp, which supported earlier findings in red sea bream (El Basuini et al., 2016) and juvenile sturgeon (Wang et al., 2016). Muscle tissue is the main edible part of the fish body (Wei et al., 2016). The nutritional contents (such as proteins and lipids) and physicochemical properties of muscle (such as shear force, pH and cooking loss) are important for assessing muscle nutritional value and muscle quality (Mir et al., 2017). In this research, we found that appropriate Cu boosted protein and lipid contents, improved shear force and pH, and reduced cooking loss in juvenile grass carp muscle. In summary, the physicochemical characteristics and nutritional value of juvenile grass carp were improved by suitable Cu level.

As mentioned before, animal growth is closely related to muscle growth and development, and collagen synthesis is closely linked to muscle growth, whereas our research demonstrated that Cu could enter the muscle, so we subsequently explored the effects of Cu on collagen synthesis and myofiber growth and development in juvenile grass carp.

### 4.2 Cu increased collagen content related to the regulation of collagen synthesis in fish muscle

Collagen makes up 3% to 10% of the protein in fish muscle (Delbarre et al., 2006). By multiplying the HYP content by 8, the collagen content can be estimated (AOAC, 2006). Meanwhile, Sirius red staining is one of the staining methods for collagen fibers, which are colored red (Hyllested et al., 2002). Our results demonstrated that appropriate Cu level increased HYP contents and collagen fibers by Sirius red staining in juvenile grass carp muscle, indicating that Cu increased the collagen content of fish muscle. Collagen content is largely influenced by collagen synthesis. In fact, collagen synthesis is closely related to transcription, translation initiation and posttranslational modifications (Gelse et al., 2003). Therefore, determining the effects of Cu on collagen transcription, translation initiation, and posttranslational modifications was the next step of our research.

Most of type I collagen was found in grass carp muscle. Two α1 chains (Col1α1) and one α2 chain (Col1α2) make up type I collagen (Sun et al., 2018). Our findings revealed that Col1α1 and Col1α2 mRNA and protein levels were elevated by suitable Cu level, and the TGF-β1/Smads signaling pathway might be responsible for this result. The TGF-β1/Smads signaling pathway regulates type I collagen transcription (Yu et al., 2019). TGF-β1 binds to its receptor, recruits, and phosphorylates Smad2 and Smad3, forms a complex with Smad4, translocate into the nucleus as a transcription factor, and binds to target genes through other transcription factors such as Sp1 to initiate collagen synthesis, thus enhancing collagen deposition (Jinnin, 2010). Our results showed that appropriate Cu level upregulated the mRNA levels of *TGF-β1*, *Smad2*, *Smad3*, *Smad4*, and *Sp1* and the protein levels of TGF-β1, p-Smad2, Smad3 and Smad4 in juvenile grass carp muscle. Further correlation analysis revealed that Col1α1 and Col1α2 protein levels were positively correlated with TGF-β1, p-Smad2, Smad3 and Smad4 protein levels (Table 5), supporting our hypothesis. TGF-β1 was elevated by Cu and might be associated with connective tissue growth factor (CTGF). CTGF acts as a cofactor of TGF-β1, which is synthesized by myoblasts and myofibers, thereby inducing of type I collagen synthesis (Kong et al., 2014). In this experiment, we found that appropriate Cu levels enhanced *CTGF* mRNA abundance in juvenile grass carp muscle, and correlation analysis revealed that *TGF-β1* and *CTGF* mRNA levels were positively associated (Table 5). In conclusion, Cu-increased muscle collagen content might be connected to the CTGF/TGF-β1/Smads signaling pathway.

**Table 5:** Correlation coefficient of collagen synthesis and myofiber growth parameters of juvenile grass carp (*Ctenopharyngodon idella*).

Independent parameters	Dependent parameters	Correlation coefficients	*P*-value
Col1α1 protein level	TGF-β1 protein level	+0.921	0.026
	p-Smad2 protein level	+0.952	0.013
	Smad3 protein level	+0.964	0.008
	Smad4 protein level	+0.974	0.005
	*LARP6* mRNA level	+0.903	0.036
Col1α2 protein level	TGF-β1 protein level	+0.925	0.024
	*LARP6* mRNA level	+0.730	0.162
	p-Smad2 protein level	+0.990	0.001
	Smad3 protein level	+0.988	0.002
	Smad4 protein level	+0.990	0.002
*TGF-β1* mRNA level	*CTGF* mRNA level	+0.912	0.031
HYP content	*LARP6* mRNA level	+0.827	0.084
Collagen content	LH activity	+0.787	0.114
	PHD activity	+0.938	0.018
	LOX activity	+0.956	0.011
LOX activity	*MyoD* mRNA level	+0.921	0.026
	MyoG protein level	+0.849	0.069
	*Myf5* mRNA level	+0.965	0.008
	*MRF4* mRNA level	+0.947	0.014
	MyHC protein level	+0.892	0.042

Col1α1 = type I collagen α1; Col1α2 = type I collagen α2; TGF-β1 = transforming growth factor-β1; HYP = hydroxyproline; LOX = lysine oxidase; *LARP6* = La Ribonucleoprotein 6; *CTGF* = connective tissue growth factor; LH = hydroxylase; PHD = proline hydroxylase; *MyoD* = myogenic differentiation; MyoG = myogenin; *Myf5* = myogenic factor 5; *MRF4* = myogenic regulatory factors 4; MyHC = myosin heavy chain.

After transcription, Col1α1 and Col1α2 translation initiation is mediated by LARP6, a member of the RNA-binding protein superfamily, which specifically binds the 5′ stem loop in *Col1α1* and *Col1α2* mRNAs (Stefanovic et al., 2022). Our results suggested that optimal dietary Cu level upregulated *LARP6* mRNA levels in juvenile grass carp muscle. Further correlation analysis showed that HYP content and Col1α1, and Col1α2 protein levels were positively correlated with *LARP6* mRNA levels (Table 5), indicating that improved collagen synthesis with optimal dietary Cu might be partly related to translation initiation. In addition to translation initiation, posttranslational modifications also play an important role in collagen synthesis. As mentioned previously, posttranslational modifications are partially influenced by PHD, LH and LOX. We found that optimal Cu enhanced PHD, LH and LOX activities in juvenile grass carp muscle, and further correlation analysis revealed a positive correlation between collagen content and PHD, LH and LOX activities (Table 5), demonstrating that improvement of collagen content by optimal dietary Cu might be related partially to its posttranslational modifications.

As mentioned earlier, collagen is the main ECM component. Lysine oxidase regulates ECM cross-linking in extracellular muscle connective tissue while promoting differentiation within myogenic progenitor cells, ultimately leading to myofiber growth and development (Gabay et al., 2020). Hence, we explored the mechanism whereby dietary Cu affects myofiber growth and development.

### 4.3 Cu promoted the myofiber growth of fish related to proliferation and differentiation of myoblasts

Fish muscle growth and development are achieved by a combination of myofiber proliferation and hypertrophy (Valente et al., 2013). As fish body weight increases, myofiber diameters increase (Yu et al., 2017). The frequency of myofibers with diameter <20 and >50 μm represents a more active growth pattern of myofiber proliferation and hypertrophy (Silva et al., 2009). In the present study, we first revealed that appropriate dietary Cu decreased the frequency of myofibers with diameter <20 μm and increased the myofiber diameters and the frequency of myofibers with diameter >50 μm in juvenile grass carp, indicating that optimal dietary Cu promoted myofiber hypertrophy in fish. Fish myofiber growth and development is closely related to myoblast proliferation (Johnston et al., 2011). Proliferation is regulated by cell cycle proteins (such as cyclin B, cyclin D and cyclin E) (Mastroyiannopoulos et al., 2012) and PCNA (Shen et al., 2003). The results of the present study first showed that suitable Cu level upregulated cyclin B, cyclin D, cyclin E and *PCNA* mRNA levels in juvenile grass carp muscle, confirming that Cu-improved fish myofiber growth and development might be connected to promoting myoblast proliferation.

In addition to proliferation, myoblast differentiation also has a crucial function in myofiber growth and development. Myogenic regulatory factors (MyoD, Myf5, MyoG and MRF4) play a key role in animal myogenic cell differentiation (Sabourin and Rudnicki, 2000). MyHC is a marker protein of late differentiation of myogenic cells (Chu et al., 2010). In this experiment, we discovered that optimal Cu level increased *MyoD*, *MyoG*, *Myf5*, *MRF4* and *MyHC* mRNA levels, as well as MyoD, MyoG and MyHC protein levels in juvenile grass carp muscle. Cu-upregulated myogenic regulators might be related to LOX. Lysyl oxidase can upregulate the mRNA levels of *MyoD*, *Myf5*, *MyoG* and *MyHC* in mouse myogenic progenitor cells (Gabay et al., 2020). Our findings revealed that appropriate Cu level increased LOX activity in juvenile grass carp muscle. The *MyoD*, *Myf5* and *MRF4* mRNA levels, as well as MyoG and MyHC protein levels were positively linked to LOX activity according to further correlation analysis (Table 5), confirming our assumption. In addition, myofiber growth was negatively regulated by MSTN, which inhibited C2C12 myogenic cell proliferation and differentiation (Langley et al., 2002). Our research revealed that suitable Cu level decreased *MSTN* mRNA levels in juvenile grass carp muscle, implying that Cu-promoted myofiber development might be associated with a decrease in *MSTN* mRNA levels.

In summary, proper Cu level promoted myofiber growth and development in fish muscle and might be influenced by myoblast proliferation and differentiation.

### 4.4 High level of Cu adversely affected the growth performance and muscle growth of fish

Compared to the optimal level group, the high Cu level (8.07 mg/kg) reduced growth performance and some of muscle collagen synthesis (except for *Smad2* and *Smad4* mRNA levels) and myofiber growth and development-related indices of juvenile grass carp, although these results were still higher than those of the control group without added Cu. These results may be partially explained by several aspects. First, our research revealed that high Cu levels reduced growth performance compared to optimal dietary Cu level, which may be related to the reduced FE in the fish. High Cu could reduce the FE of fish, thus leading to reduced growth performance (Wang et al., 2016). Second, the reduction in muscle collagen synthesis by high Cu level in juvenile grass carp may be connected to certain Cu-dependent enzymes. This study found that serum Cp activity was reduced when dietary Cu was too high. Cp is involved in iron metabolism by having iron oxidase activity, converting Fe^2+^ to Fe^3+^ (Roeser et al., 1970). The reduction of its activity caused iron deposition (Vulpe et al., 1999), and excessive iron content caused downregulation of *TGF-β1* mRNA levels (Guo et al., 2017). This might result in the downregulation of signaling molecules that regulate collagen transcription, ultimately leading to a decrease in collagen transcription-related indices. Third, the decrease in myofiber growth and development indices caused by high Cu level may be connected to selenium deficiency. One study revealed that high Cu levels resulted in lower whole-body selenium content in Atlantic salmon (Berntssen et al., 1999), and dietary selenium deficiency decreased the growth and development of rainbow trout myofibers (Wang et al., 2021). More research should be conducted regarding the specific mechanism.

### 4.5 Requirements and the comparison between CuCit and CuSO_4_·5H_2_O of fish

**Fig. 4:**
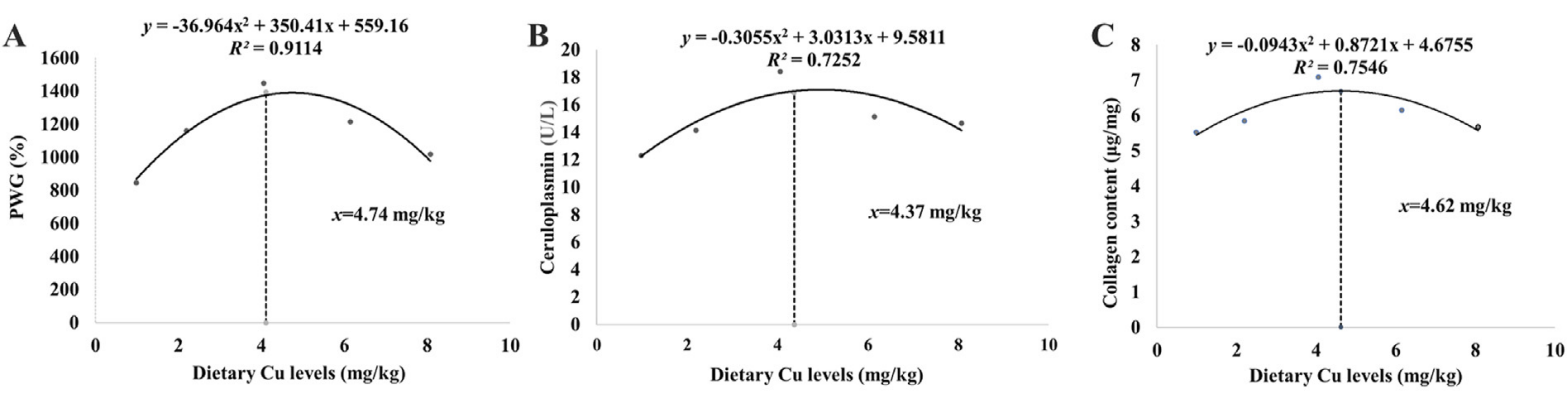
Quadratic regression analysis of (A) percentage weight gain (PWG), (B) ceruloplasmin activity, and (C) collagen content in juvenile grass carp fed diets with various Cu levels for 9 weeks.

In accordance with the quadratic regression analysis of PWG, serum Cp and collagen content in juvenile grass carp muscle, Cu requirements (CuCit as Cu source) were estimated at 4.74, 4.37 and 4.62 mg/kg diet, respectively (Fig. 4). These results revealed that the Cu requirements of several indicators were comparable. This maybe because many Cu-dependent enzymes use Cu as a cofactor or variable component in fish, which should be given at relatively low quantities to balance nutritional requirements. Additionally, earlier research in our lab on the requirements for other trace elements, such as the requirements for iron based on hematological characteristics (serum iron and hemoglobin), was also comparable to the requirements of juvenile grass carp for growth (Zhang et al., 2016). These findings imply that trace elements for fish health should be utilized with caution.

In addition, the efficacy of CuCit against CuSO_4_·5H_2_O were 131.80% and 115.38% based on PWG and serum Cp activity in juvenile grass carp muscle, respectively. These findings revealed that CuCit was superior to CuSO_4_·5H_2_O. A possible mechanism may be connected to different absorption rates and salt ions between CuCit and CuSO_4_·5H_2_O. The molecules of organic Cu are electrically neutral and are not subject to charge in the intestinal tract, which does not form absorption resistance or deposition phenomena, resulting in rapid absorption and the reduction of many biochemical processes, saving physical energy expenditure and high bio-efficiency (Ashmead, 1991).

## 5 Conclusion

In conclusion, the present study revealed that dietary Cu increased growth performance and improved the nutritional status of Cu in fish tissues. Furthermore, we first found that appropriate dietary Cu level (4.06 mg/kg) regulates muscle growth and development, which might be related to: (1) elevating collagen content, which might be attributed to the activation of the CTGF/TGF-β1/Smads signaling pathway, upregulating of *LARP6* mRNA levels, and enhancement of the activities of related enzymes (PHD, LH and LOX) for collagen synthesis; (2) promoting proliferation and differentiation of myogenic cells, leading to myofiber growth and development. Finally, based on PWG, serum Cp activity and collagen content quadratic regression analysis in juvenile grass carp muscle, the Cu requirements (CuCit as Cu source) were identified as 4.74, 4.37 and 4.62 mg/kg diet, respectively. Based on PWG and serum Cp activity in juvenile grass carp muscle, compared to CuSO_4_·5H_2_O, the efficacy of CuCit were 131.80% and 115.38%, respectively.

## Author contributions

**Rui Ma:** Manuscript writing, Formal analysis; **Lin Feng:** Methodology, Supervision; **Pei Wu, Yang Liu:** Methodology; **Shu-Wei Li, Ling Tang, Cheng-Bo Zhong, Dong Han, Wen-Bing Zhang:** Resources; **Hong-Mei Ren, Jia-Yong Tang:** Management; **Xiao-Qiu Zhou:** Writing - review & editing, Funding acquisition, Project administration, Supervision; **Wei-Dan Jiang:** Conceptualization, Supervision. **Wei-Dan Jiang** had primary responsibility for the final content of the manuscript. All authors carefully read and approved the final revision of the manuscript.

## Declaration of competing interest

We declare that we have no financial and personal relationships with other people or organizations that can inappropriately influence our work, and there is no professional or other personal interest of any nature or kind in any product, service and/or company that could be construed as influencing the content of this paper.
