# Supplementary material for: A new insight on copper: Promotion of collagen synthesis and myofiber growth and development in juvenile grass carp (Ctenopharyngodon idella)
Source: Anim Nutr. 2023 Jul 8;15:22–33. doi: 10.1016/j.aninu.2023.06.009 (PMC10522946; doi:10.1016/j.aninu.2023.06.009)
Supplement: Multimedia component 1 [file mmc1.docx]

**Table S1**

Real-time PCR primer sequences.

| Gene | Primer sequence forward | Primer sequence reverse | Accession number |
| --- | --- | --- | --- |
| *ATP7A* | CCGTTATCGCAGGCTCTAT | TGTGCTTCCTCCACCAGTT | MK770648.1 |
| *Col1α1* | CAACAGCCGCTTCACATACA | GGCGATGTCAATAATAGGCAG | HM363526.1 |
| *Col1α2* | CAAGAACAGCATCGCCTACAT | AGATGGTTTATTCGTTCTGTATTCA | HM771241.1 |
| *TGF-β1* | TTGGGACTTGTGCTCTAT | AGTTCTGCTGGGATGTTT | EU099588.1 |
| *CTGF* | CAAGCTGAGAGAAGGTGGAA | CAGGAGGAGCCAGATAGTCA | KY024218.1 |
| *Smad2* | GTCCTCCATCTTGCCTTTCAC | CTTCTCGCACCATTTCTCCTC | DQ912858.1 |
| *Smad3* | ATTGAGCCTCCGAGCAACTAT | GAAAGATTTGGGGAACCTGTG | DQ912859.1 |
| *Smad4* | ATCACCGCCATCACCACTAAC | TATTCCTGGGGACACCACTCT | HQ596213.1 |
| *Sp1* | TTCTGCGGGAAACGTTTCAC | ACTACGCATGAACCGTTTGG | KY081668.1 |
| *LARP6* | CTGAGGAGTGTGCCATCGTAG | TTCTTGGGAGGTTTGGTGCC | OL438919 |
| *cyclin B* | TTATTGACTGGCTTGTGC | GAGCTGCTTCTTTGGAAC | KX085204.1 |
| *cyclin D* | TCAGTGACACCGCACGAT | GCACAGAGGGCCACAAAT | XM_051889963.1 |
| *cyclin E* | ACTTGGGTCAGGACTACTTTG | AGCGATGAAGAGACAGGAGA | KX682385.1 |
| *PCNA* | CCTACCGCTGCGACAGAAACCT | TCAAAGACAAGAGCCAAAGAGT | XM_051860319.1 |
| *MyoG* | AGAGGAGGTTGAAGAAGGTC | GTTCCTGCTGGTTGAGAGA | JQ793897 |
| *MyoD* | CCCTTGCTTCAACACCAACG | TCTCCTCTCCCTCATGGTGG | GU218462 |
| *Myf5* | GGAGAGCCGCCACTATGA | GCAGTCAACCATGCTTTCAG | GU290227 |
| *MyHC* | ACGCTCATCACCACCAACCC | CAGCCTCCTCTGTGCCATCA | EU414733.1 |
| *MRF4* | GAAAATCTGCTCCAACCGA | CGCTGCGTAAAATCTCCA | JQ793896 |
| *MSTN* | GAACAGGCTCCGAACATCAGC | GGTTGCAGTCCTTCTTCTCC | KM874827 |

*ATP7A* = Cu-transporting ATPase 1; *Col1α1* = type I collagen α1; *Col1α2* = type I collagen α2; *TGF-β1* = transforming growth factor-β1; *CTGF* = connective tissue growth factor; *Sp1* = Specificity protein 1; *LARP6* = La Ribonucleoprotein 6; *PCNA* = proliferating cell nuclear antigen; *MyoD* = myogenic differentiation; *MyoG* = myogenin; *Myf5* = myogenic factor 5; *MRF4* = myogenic regulatory factors 4; *MyHC* = myosin heavy chain; *MSTN* = myostatin.
